# Associations of social and economic and pregnancy exposures with blood pressure in UK White British and Pakistani children age 4/5

**DOI:** 10.1038/s41598-018-27316-1

**Published:** 2018-06-12

**Authors:** Jane West, Debbie A. Lawlor, Gillian Santorelli, Paul Collings, Peter H. Whincup, Naveed A. Sattar, Diane Farrar, John Wright

**Affiliations:** 1 Bradford Institute for Health Research, Bradford, UK; 2 MRC Integrative Epidemiology Unit at the University of Bristol, Bristol, UK; 3 Population Health Science, Bristol Medical School, Bristol, UK; 4 Population Health Research Institute, St George’s, University of London, London, UK; 5 Institute of Cardiovascular and Medical Sciences, BHF Glasgow Cardiovascular Research Centre, University of Glasgow, Glasgow, UK

## Abstract

South Asians have higher rates of coronary heart disease (CHD) than White European individuals. Blood pressure (BP) is one of the most important risk factors for CHD and ethnic differences in BP have been identified in childhood. Early life exposures could explain some of these differences. We examined associations of family social and economic and maternal pregnancy exposures and BP at age 4/5 in 1644 White British and 1824 Pakistani mother-offspring pairs from the Born in Bradford study. We found that systolic BP was similar but diastolic BP was higher, in Pakistani compared to White British children (adjusted mean differences were −0.170 mmHg 95% CI −0.884, 0.543 for systolic BP; 1.328 mmHg 95% CI 0.592, 2.064 for diastolic BP). Social and economic exposures were not associated with BP in either ethnic group. Maternal BMI was positively associated with BP in both groups but this association was mediated by child BMI. Only gestational hypertension was associated with child systolic and diastolic BP and this was only identified in Pakistani mother-offspring pairs. These findings suggest that Pakistani populations may have a different BP trajectory compared to White British groups and that this is already evident at age 4/5 years.

## Introduction

South Asians have higher rates of coronary heart disease (CHD) than White European individuals^1^. High blood pressure (BP) is one of the most important risk factors for CHD^2^ and higher levels of adiposity are a key risk factor for elevated BP^3^. One reason that South Asians are hypothesized to have higher CHD risk is that for a given body mass index (BMI), they have higher fat mass and lower lean mass^4^. This has led to the suggestion that they have a specific thin-fat phenotype and there is some evidence that this is present from birth and may have a developmental origin^5–8^.

A small number of studies have shown ethnic differences in BP in children, in particular lower mean systolic and higher mean diastolic blood pressure in UK South Asian children compared to White British children at age 9/10^9^ and also through adolescence^10^. This lower systolic BP in South Asians is somewhat surprising given evidence that we and others have shown greater fat mass in South Asians from birth and through childhood^8,11^. Furthermore, there is some evidence that in adulthood, BP (systolic and diastolic) is higher in South Asians compared to White Europeans^12^, and also evidence that (certainly in White Europeans) both systolic and diastolic BP in childhood tracks and is associated with BP in adulthood^13^. Given the small number of studies that have looked at childhood ethnic differences in BP, the lower childhood and adolescent systolic BP in South Asians may be a chance finding or it is possible that what is assumed to be a fat mass driven association between body mass index and higher BP is also driven by lean mass. Other potential explanations include that South Asian BP trajectories across the life course have different patterns to those in White Europeans (leading to differences in associations/tracking across the life course), that there are other early life drivers (beyond childhood fat and lean mass) of BP differences between South Asian and White British children, and/or that the early life drivers of systolic and diastolic BP differ. Specifically, maternal exposures could explain some of these ethnic differences in BP. For example, several studies in largely European origin populations suggest an association between maternal BMI and offspring systolic^14^ and diastolic BP^15^. There is also some evidence that gestational diabetes (GDM) is associated with offspring hypertension in Eastern Asian children^16^ and is associated with higher systolic BP in South Asian children^17^. Furthermore, whilst there are inconsistent reports of associations between social and economic background and BP in European origin children^9,10^, little is known about how these characteristics relate to BP in South Asian children despite known differences in the distributions of social and economic characteristics and how they relate to childhood adiposity and other health related outcomes, between White European and South Asian populations^18,19^.

Our research objectives were to determine:

- Whether the distributions of systolic and diastolic BP at age 4/5 differed between White British and Pakistani origin children all born and growing up in the same UK city.
- The magnitude of any associations of family social and economic and maternal pregnancy exposures with offspring BP in these two ethnic groups.
- Whether any associations of these early life exposures with offspring BP differed in their magnitude or direction between the White British and Pakistani mother-offspring pairs or between systolic and diastolic BP.

The specific maternal exposures that we examined were educational attainment, as well as family housing tenure and receipt of benefits (family social and economic exposures), and maternal BMI, smoking in pregnancy, fasting and postload glucose, GDM, hypertensive disorders of pregnancy (HDP) including gestational hypertension and pre-eclampsia (maternal pregnancy exposures).

## Materials, Subjects and Methods

### Participants

**Figure 1:**
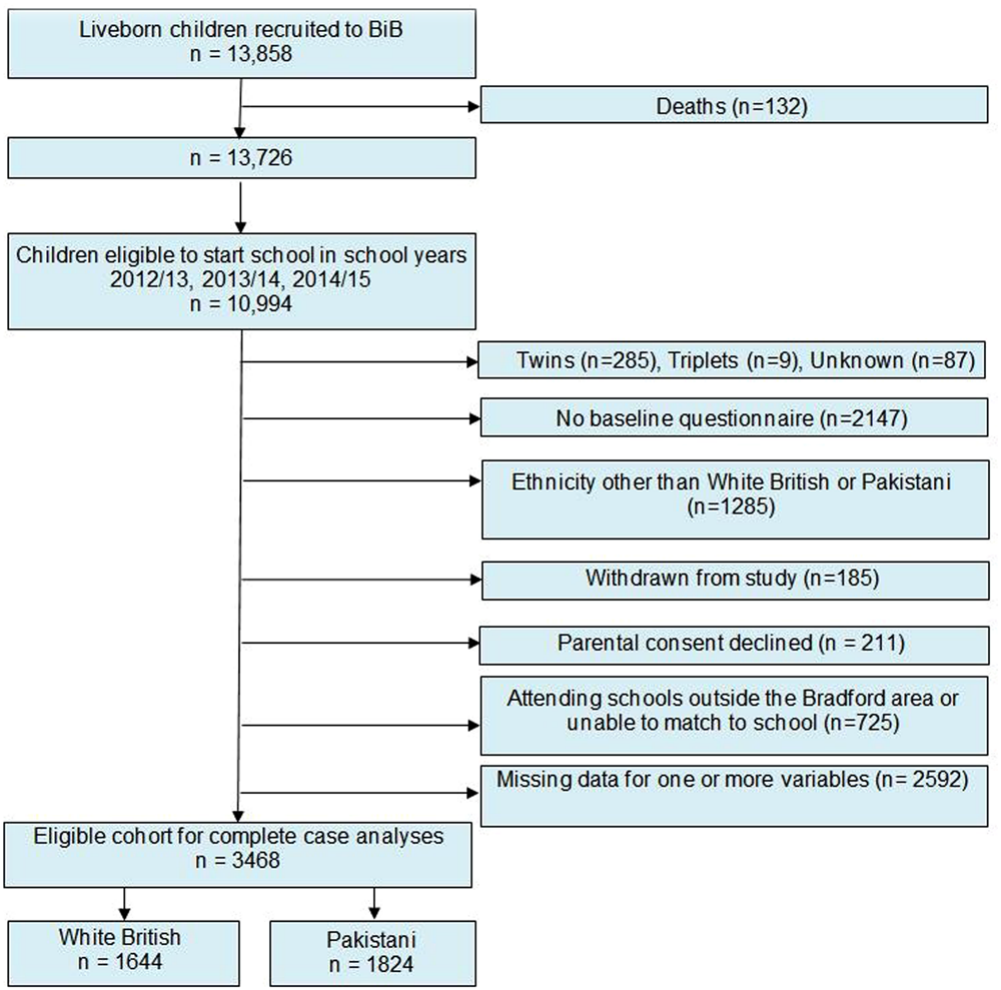
Flow chart of study sample.

The Born in Bradford (BiB) cohort study is a prospective pregnancy and birth cohort based in the 6^th^ largest city in the UK: Bradford in the North of England. Full details of the study methodology have been previously reported^20^. Briefly, to be eligible for the study women had to attend antenatal booking clinic between March 2007 and December 2010 and be booked to give birth in the city of Bradford. A total of 13818 liveborn children were recruited to the study. There were 132 child deaths and of the remaining 13, 726, 10,999 were eligible to start school in the school years 2012/13; 2013/14; 2014/15. Parents of eligible children were mailed information about the blood pressure measurements 8 weeks prior to the scheduled measurements with an ‘opt out’ consent form and pre-paid envelope should they wish to withdraw their child from the measurements. The opt-out consent is consistent with the consent process used for the NCMP and was approved by the National Health Service research ethics committee. Ethnic groups other than Pakistani and White British were excluded because they included too few participants within each group for meaningful analyses. We also excluded those with no baseline questionnaire (because of recruitment later than the antenatal OGTT recruitment time), twins and triplets and those who had withdrawn from the study (Fig. 1). Parental consent was refused for 211 (3%) children with similar proportions in each ethnic group (White British 2.6% Pakistani 3.4%) and 725 (10% of all eligible participants) could not be matched to their school or were attending a school outside the Bradford district (15% White British and 6%% Pakistani). A further 2592 were excluded if data were missing for one or more variables, of these 47% were White British and 53% Pakistani origin. The remaining 3468 mother-offspring pairs (1644 White British and 1824 Pakistani) form the sample for this study. Ethics approval for the study was granted by Bradford National Health Service Research Ethics Committee (ref 06/Q1202/48).

### Assessment of ethnicity

Ethnicity was self-reported by mothers at the recruitment interview and based on UK Office of National Statistics guidance details of which have been previously reported^21^.

### Social and economic measures

Information on social and economic indicators (education, receipt of benefits, housing tenure) was obtained from the interview with the woman at recruitment. We equivalised the mother’s highest educational qualifications (based on the qualification received and the country obtained) as previously reported^21^. Receipt of means tested benefits was based on the mother or her household receiving any of: Income Support, Job Seekers Allowance, Working Tax Credit or Housing Benefit. Housing tenure was categorised according to whether the woman lived in a household where the home was either part-owned (i.e. mortgaged) or owned outright, or not (i.e. rented). Information on smoking was obtained at the questionnaire interview, with women categorised as having smoked cigarettes at any stage of their pregnancy or not.

### Maternal pregnancy measurements

Height was measured (unshod and in light clothing) at recruitment (26–28 weeks gestation) using a Leicester Height Measure. Weight at first antenatal clinic assessment when women were median 12 weeks (IQR 11, 14) was abstracted from the antenatal records and was used with height measured at recruitment to calculate the woman’s early pregnancy BMI. Smoking in pregnancy was obtained from the recruitment interview. All women booked for delivery in Bradford are offered a 75 g oral glucose tolerance test (OGTT) comprising fasting and 2 hour post-load samples, at around 26–28 weeks gestation. Plasma glucose levels were assayed immediately after sampling at the biochemistry department of Bradford Royal Infirmary using the glucose oxidase method on Siemen’s Advia 2400 chemistry autoanalysers and Siemen’s Advia Centaur assay. GDM was defined according to modified WHO criteria operating at the time these women were pregnant as either fasting glucose ≥6.1 mmol/l or 2 h glucose ≥7.8 mmol/l^21^. Women were classified as having gestational hypertension if they had a systolic measure ≥ 140 and a diastolic ≥ 90 mmHg on 2 or more occasions after 20 weeks gestation and pre-eclampsia if significant proteinuria (>1+) accompanied hypertension; information on this was obtained from the antenatal records.

### Offspring measurements

BP measurements were collected by school nurse teams who visited 143 primary schools in Bradford. Measurements were recorded using Omron HEM-907 electronic monitors and were collected at the same time as skinfold thickness measurements^22^ all according to a written protocol. The appropriate cuff size (either child or small adult) was used. Children were seated for 2 minutes prior to the BP measurement and all measures were recorded using the left arm. We recorded one BP measurement consistent with other studies undertaken within a school setting^23,24^. This was a pragmatic approach to data collection with the aim of minimising discomfort to children who were aged just 4 or 5, and limiting any disruption to teaching.

### Other variables

Information on maternal age was obtained at the recruitment interview, and information on parity, gestational age, sex of the child was abstracted from medical records. Age at BP measurement was calculated using date of birth and date of measurement which were recorded in school when the BP measurement was taken. Our focus here is on maternal characteristics that could be modified before or during pregnancy and that potentially impact on offspring blood pressure in childhood. Whilst birth weight has been shown to be associated with offspring blood pressure, this is unlikely to be a direct causal effect but rather birth weight acts a proxy for maternal risk factors that might – through developmental origins processes – impact child outcomes. Furthermore, it and gestational age at delivery are not readily modifiable.

### Statistical analyses

**Figure 2:**
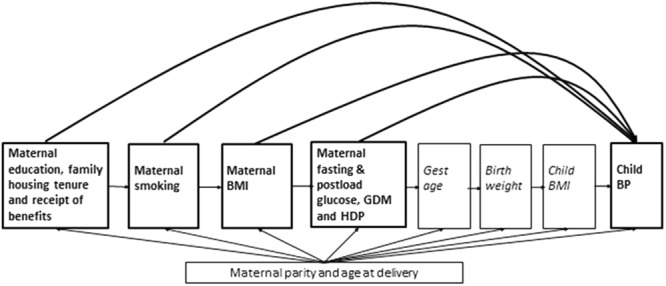
Conceptual model for selecting confounders. Notes: (**a**) All arrows indicate that we believe it is plausible that the characteristic(s) at the base of the arrow influence the characteristic(s) at the head of the arrow. The associations that we focus on here are maternal characteristics that could be modified during or before pregnancy and their potential impact on offspring blood pressure in childhood (highlighted in bold and heavily weighted boxes and arrows). (**b**) This is not a complete graph of all plausible relations between all of the characteristics shown; it is an illustration of our conceptual model used to decide what to consider for each of the maternal early life risk factors (the first 4 left side boxes) for child BP that we considered to be confounders (i.e. influencing the risk factor and child BP). Thus, for e.g. for maternal smoking in the best confounder adjusted model we adjusted for maternal parity, age at delivery, education and family housing tenure and receipt of benefits, but not other characteristics.

All analyses were performed using STATA/SE software (Stata/SE 12 for Windows, StataCorp LP, College Station, TX, USA). Distributions of maternal and child characteristics including their systolic and diastolic BP, by ethnicity and sex are presented using numbers (%) for categorical characteristics and mean (SD) or median (IQR) for continuously measured variables. Unadjusted and sex and age only adjusted differences in mean systolic and diastolic BP between White British (reference) and Pakistani children were determined using linear regression. Multivariable linear regression was used to examine the associations of maternal BMI, fasting and post-load glucose, GDM, HDP (gestational hypertension and pre-eclampsia), smoking in pregnancy, maternal education, housing tenure and receipt of benefits with offspring systolic and diastolic blood pressure at age 4/5 within each ethnic group. Differences in the magnitudes or directions of associations were explored by looking at the ethnic specific point estimates and including an interaction term between ethnicity and the exposures for each of these associations. Potential confounders for each exposure were selected a priori based on existing published literature and our previous BiB analyses^8,25,26^, and avoided the bias that can be generated with all exposures are mutually adjusted for each other^27^. Figure 2 shows our conceptual model for how the key risk factors are plausibly related to each other and how other characteristics might be confounders. We used this model to adjust for potential confounders. For all of the associations in model 1, we adjusted for child sex and age (in months) at BP measurement. Whilst we acknowledge that child sex and age at BP measurement could not confound the association as these are unlikely to be able to influence the early life exposures we have examined, they were included to reduce child BP variability. The adjustments that were further included varied for each risk factor (Fig. 2) and are shown in Table 1.

**Table 1:** Covariables adjusted for in each model.

Risk factors	Covariables adjusted for^*^			
	Model 1	Model 2 - as Model 1 with additional adjustment for:	Model 3 - as Model 2 with additional adjustment for:	Model 4 - as in Model last model in row plus
Maternal education	Child sex Age at BP measurement			Child BMI at age when BP measured
Family housing tenure	Child sex Age at BP measurement			Child BMI at age when BP measured
Family receipt of benefits	Child sex Age at BP measurement			Child BMI at age when BP measured
Maternal BMI	Child sex Age at BP measurement	Maternal age at delivery Parity Maternal pregnancy smoking Maternal education Family housing tenure Family receipt of benefits		Child BMI at age when BP measured
Maternal pregnancy smoking	Child sex Age at BP measurement	Maternal age at delivery Parity Maternal education Family housing tenure Family receipt of benefits		Child BMI at age when BP measured
Maternal fasting & postload glucose	Child sex Age at BP measurement	Maternal age at delivery Parity Maternal pregnancy smoking Maternal education Family housing tenure Family receipt of benefits	Maternal BMI	Child BMI at age when BP measured
GDM	Child sex Age at BP measurement	Maternal age at delivery Parity Maternal pregnancy smoking Maternal education Family housing tenure Family receipt of benefits	Maternal BMI	Child BMI at age when BP measured
Maternal HDP	Child sex Age at BP measurement	Maternal age at delivery Parity Maternal pregnancy smoking Maternal education Family housing tenure Family receipt of benefits	Maternal BMI	Child BMI at age when BP measured

^*^Models 1 to 3 are confounder adjusted models (though we note that child sex and age at BP measurement are not confounders; these were adjusted for to reduce BP variability). Confounders are defined as characteristics that could influence the risk factor of interest and child BP. Model 4 is an attempt to explore whether child BMI mediates any associations of early life risk factors with child BP. For this model child BMI is added to the most complete confounder adjusted model (i.e. the last one in the row for each risk factor). Figure 2 shows our conceptual model for determining which factors to consider confounders.

## Results

**Figure 3:**
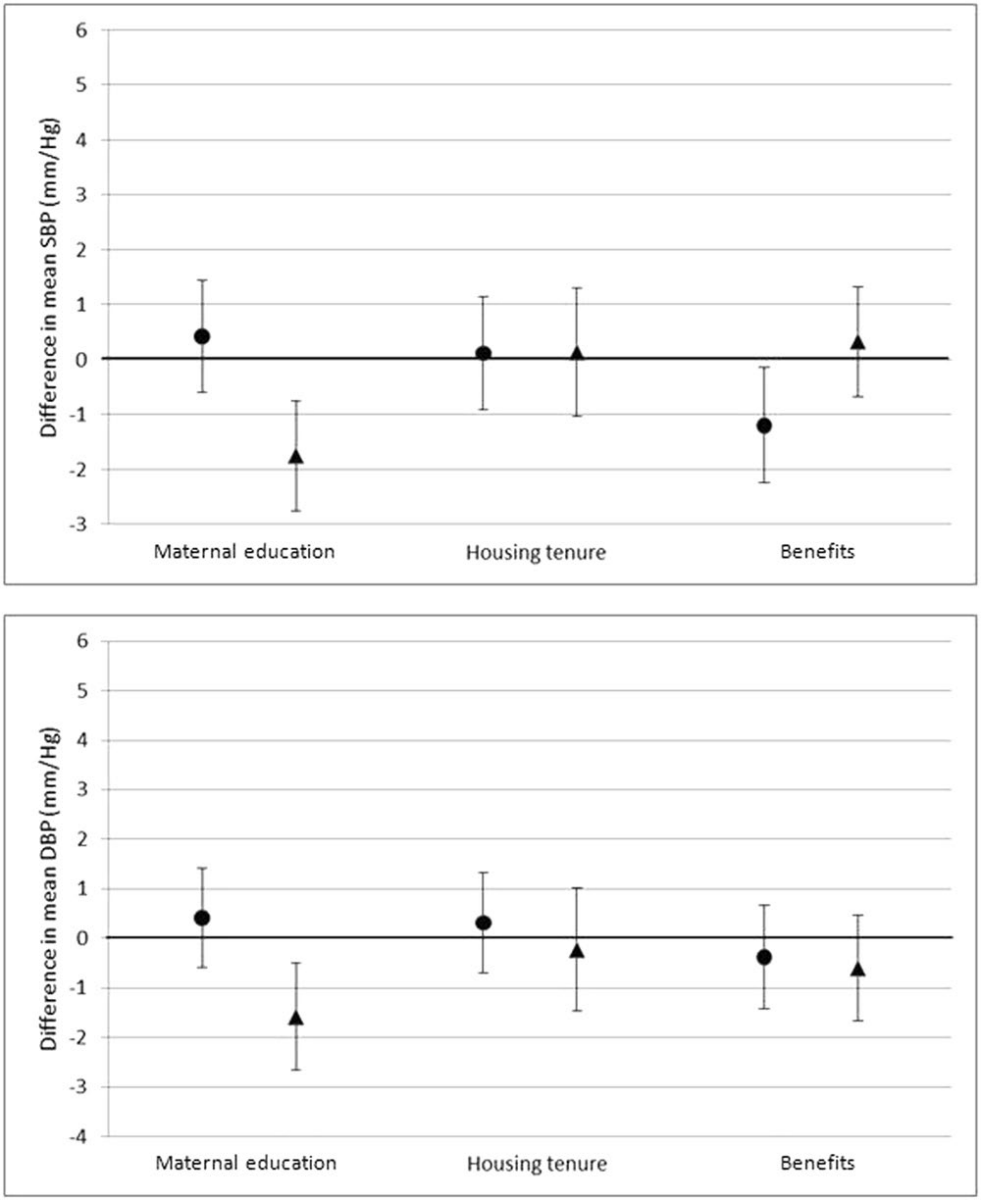
Associations between family social and economic exposures and offspring BP at age 4/5 (Model 1). ●White British ▲Pakistani. *Model 1: Adjusted for sex; age at measurement. Values are differences in means (95% CI) of outcome per maternal exposure unit or category.

Distributions of maternal and offspring characteristics for the whole cohort and by ethnicity are presented in Table 2. Maternal education was similar in both ethnic groups but more Pakistani women owned or part-owned their own home and a higher proportion received means tested benefits compared to White British women. On average, early pregnancy BMI was lower in Pakistani women and only 3% smoked during pregnancy compared to 33% of White British women. Fasting and post-load glucose levels were higher and GDM was markedly more common in Pakistani mothers compared to White British mothers (9.81% and 4.87% respectively). By contrast, around twice as many White British women had HDP (gestational hypertension) but rates of pre-eclampsia were similar in both groups. Pakistani children were taller and lighter with a lower mean BMI than White British children. Systolic BP was similar in children from both ethnic groups, but diastolic BP was on average higher in Pakistani compared with White British children. The child sex and age adjusted mean differences comparing Pakistani to White British (reference) in BP were −0.170 mmHg (95%CI: −0.884, 0.543) for systolic BP and 1.328 mmHg (95%CI: 0.592, 2.064) for diastolic BP. Supplementary Table 1 additionally shows the offspring characteristics differences by sex as well as ethnicity. Boys were taller and heavier than girls in both ethnic groups, mean BMI was similar for boys and girls. There were small sex differences in systolic and diastolic BP between boys and girls in each ethnic group and the ethnic differences in both were similar for both boys and girls. Associations between family social and economic characteristics and BP outcomes were mostly consistent with the null hypothesis in both the unadjusted (Supplementary Table 2a) and age and sex adjusted models (Model 1: Fig. 3 and Supplementary Table 3a) apart from maternal education for which there was statistical evidence for a negative association in Pakistani children for both systolic and diastolic BP. Additional adjustment for child BMI at the time of BP measurement did not markedly alter results (Supplementary Table 6a).

**Table 2:** Distributions of maternal and offspring characteristics stratified by ethnicity.

Characteristic	All *n* = *3468*	White British *n* = *1644*	Pakistani origin *n* = *1824*	p-value*
Maternal age at delivery mean(sd)	27.44 (5.57)	27.15 (6.06)	27.70 (5.07)	0.003
**Parity n (%)**				
0 1 2 3 4 or more	1349 (39) 1052 (30) 599 (17) 286 (8) 182 (5)	763 (46) 560 (34) 208 (13) 78 (5) 35 (2)	586 (32) 492 (27) 391 (21) 208 (11) 147 (8)	<0.001
**Maternal education n (%)**				
5 GCSEs or less A Level/equivalent or more	1940 (55.94) 1528 (44.06)	895 (54.44) 749 (45.56)	1045 (57.29) 779 (42.71)	0.091
**Housing tenure n (%)**				
*Owns/part owns* *Rents*	2276 (65.63) 1192 (34.37)	924 (56.20) 720 (43.80)	1352 (74.12) 472 (25.88)	<0.001
Receipt of benefits n (%)	1467 (42.30)	609 (37.04)	858 (40.47)	<0.001
Maternal BMI (kg/m^2^) mean (sd)	26.01 (5.71)	26.75 (5.97)	25.36 (5.39)	<0.001
Smoked in pregnancy n (%)	613 (17.68)	548 (33.33)	65 (3.06)	<0.001
**Maternal glucose (mmol/L) mean (sd)**				
*Fasting* *Post-load*	4.50 (0.52) 5.66 (1.48)	4.40 (0.43) 5.48 (1.29)	4.59 (0.57) 5.82 (1.61)	<0.001 <0.001
**Maternal GDM**				
n (%)	259 (7.47)	80 (4.87)	179 (9.81)	<0.001
**Maternal HDP n (%)**				
*Gestational hypertension* *Pre-eclampsia*	263 (7.58) 88 (2.54)	182 (11.07) 45 (2.74)	81 (4.44) 43 (2.36)	<0.001 <0.001
Child Height (cm) mean (sd)	108.41 (4.98)	108.09 (4.98)	108.69 (4.96)	<0.000
Child Weight (kg) mean (sd)	18.98 (3.08)	19.09 (2.82)	18.89 (3.29)	0.054
Child BMI (kg/m^2^) mean (sd)	16.08 (1.73)	16.28 (1.55)	15.90 (1.86)	<0.001
Child systolic BP mean (sd)	97.86 (10.73)	97.94 (10.56)	97.79 (10.89)	0.679
Child diastolic BP mean (sd)	61.24 (11.07)	60.51 (10.42)	61.90 (11.59)	<0.001

^*^Difference between White British and Pakistan.

**Figure 4:**
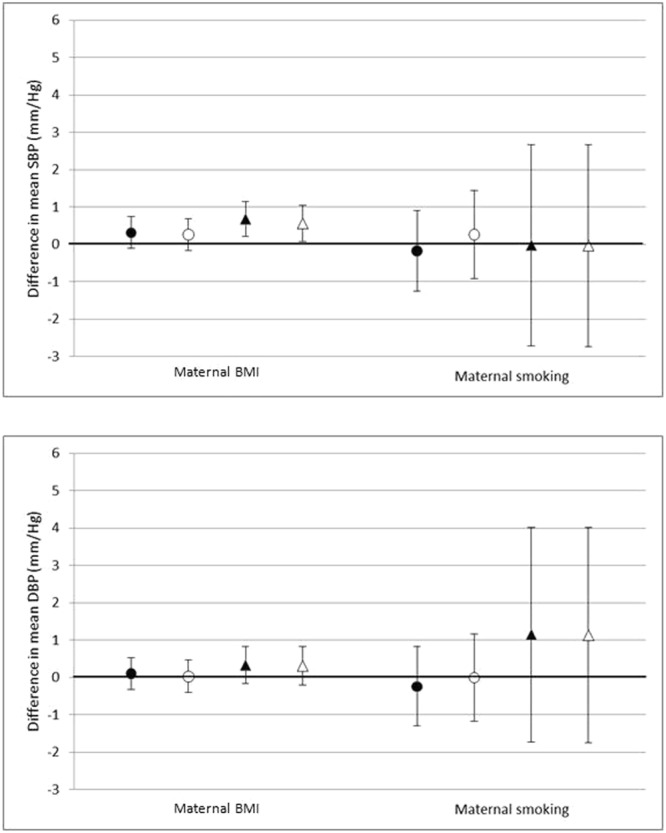
Associations between maternal pregnancy characteristics (BMI and smoking) and offspring BP at age 4/5 (Models 1 and 2). ●White British ▲Pakistani Filled shapes on graph (○): model 1; Clear dots on graph (●): model 2. *Model 1: Adjusted for sex; age at measurement; *Model 2: Adjusted for sex; age at measurement; maternal age; parity; maternal education; family housing tenure; family receipt of benefits. Maternal BMI model 2 additionally adjusted for smoking in pregnancy. Values are differences in means (95% CI) of outcome per maternal exposure unit or category, maternal BMI is difference in means per 5 kg/m^2^.

There was no strong evidence of any association between maternal BMI and systolic and diastolic BP in both groups with the exception of systolic BP in Pakistani children for which there was statistical evidence for higher systolic BP with increasing maternal BMI in all models. This association reduced slightly in the full confounder adjusted model (model 2) compared to the age and sex adjusted model (model 1) but remained positive (Fig. 4 and Supplementary Tables 2b, 3b and 4a). Further adjustment for child BMI at age of measurement reversed all associations with both systolic and diastolic BP in both ethnic groups such that they became negative (previously positive) although these associations were weak and remained close to the null hypothesis (Supplementary Table 6b). Maternal smoking in pregnancy was not associated with systolic or diastolic BP in either ethnic group, very few Pakistani women smoked during pregnancy resulting in wide confidence limits (Supplementary Tables 2b, 3b, 4a and 6b).

**Figure 5:**
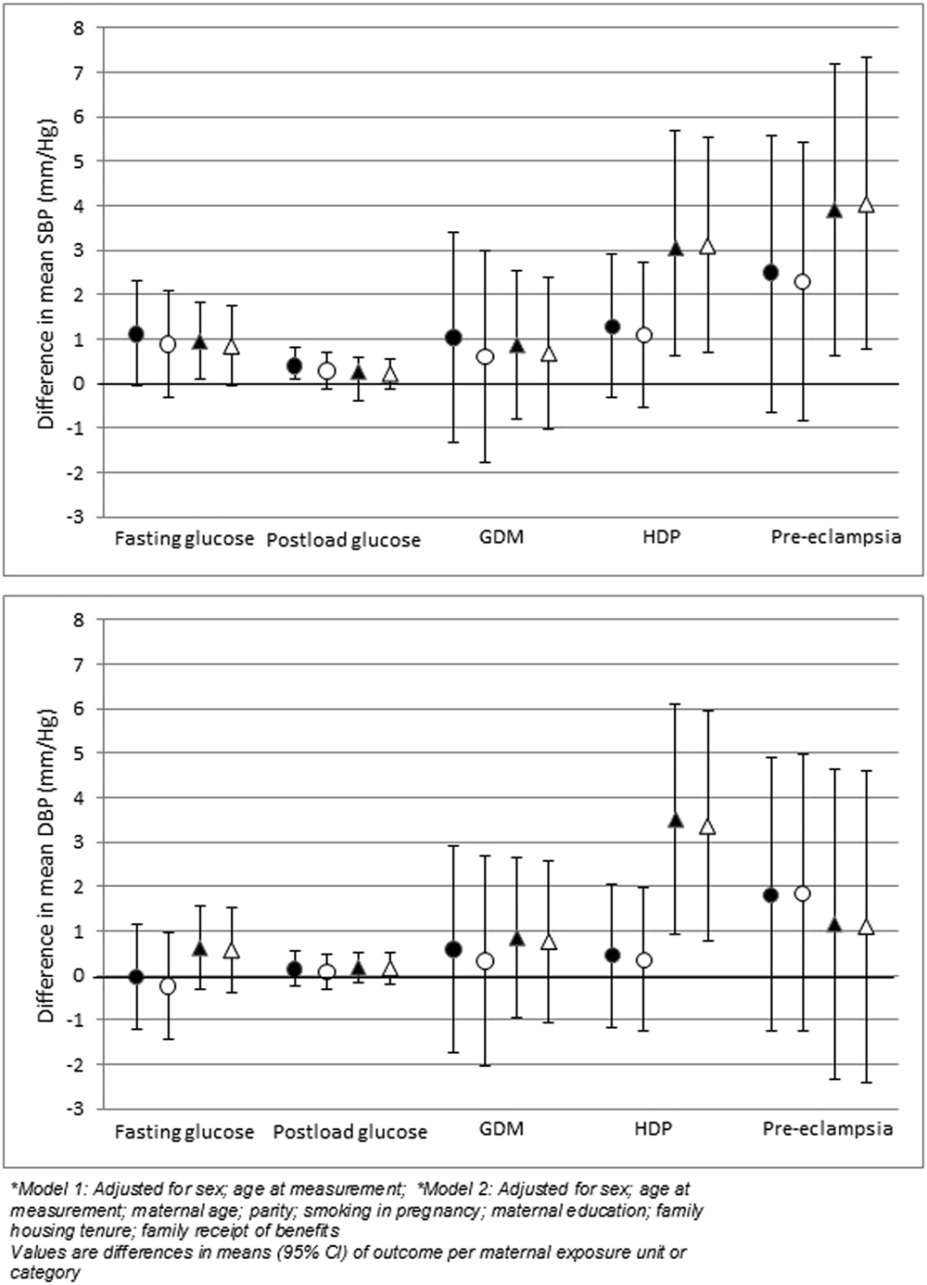
Associations between maternal pregnancy characteristics (glycaemia and HDP) and offspring BMI at age 4/5 (Models 1 and 2). ●White British ▲Pakistani. Filled shapes on graph (○): model 1; Clear dots on graph (●): model 2. *Model 1: Adjusted for sex; age at measurement; *Model 2: Adjusted for sex; age at measurement; maternal age; parity; smoking in pregnancy; maternal education; family housing tenure; family receipt of benefits. Values are differences in means (95% CI) of outcome per maternal exposure unit or category.

Maternal gestational fasting glucose, post-load glucose and GDM were positively associated with systolic BP in both groups. The associations with diastolic BP were also positive albeit smaller, with the exception of fasting glucose and diastolic BP for which the association was negative in White British children. Most of these associations were weak although there was statistical evidence for a positive association between fasting glucose and systolic BP in Pakistani children with adjustment for sex and age at measurement (model 1: Fig. 5 and Supplementary Table 3c). In the full confounder adjusted model (model 2) this association was slightly weaker and with additional adjustment for maternal BMI reduced further (model 3: Supplementary Table 5). Adjustment for child BMI at age of measurement (model 4: Supplementary Table 6c) did not alter results. HDP (gestational hypertension) was positively associated with higher systolic and diastolic BP in both groups but the magnitude of these differences and the statistical evidence were each markedly stronger in Pakistani compared to White British children. This was the case in age and sex adjusted (model 1) and full confounder adjusted (model 2) models (Fig. 5 and Supplementary Tables 3c and 4b). The interaction term suggests that the effect of HDP (gestational hypertension) varies by ethnicity (Supplementary Tables 3c and 4b). These associations remained strong following further adjustment for maternal BMI (model 3: Supplementary Table 5) and child BMI (model 4: Supplementary Table 6c). Associations between HDP (pre-eclampsia) offspring outcomes were also positive but with wide confidence limits that mostly included the null value and reflected the smaller number of participants with pre-eclampsia (Fig. 5 and Supplementary Tables 3c and 4b).

## Discussion

Using the BiB cohort, we have previously reported ethnic differences in size and adiposity in particular, how Pakistani children are taller and lighter and have a lower BMI than White British children at age 4/5^26^. In these analyses, we now show that ethnic differences in BP are also present at this age. Specifically, we find that Pakistani children have a similar systolic BP but higher diastolic BP compared to White British children. This is consistent with most previous reports of ethnic differences in child and adolescent BP^9,10,28^ but not all^29^. Higher diastolic BP in Pakistani children was seen in analyses of Health Survey for England data for children aged between 5 and 15^28^. The Child Health and Heart Study in England (CHASE) reported lower systolic but higher diastolic BP in South Asian schoolchildren compared to White European children at age 9–11^9^ and the Determinants of Adolescent Social Well-being and Health Study (DASH) showed that mean diastolic BP was higher among South Asians in adolescence (age 14–16) compared to White British children^10^ although this difference was not seen at age 11–13^30^. By contrast, a much smaller study of UK Pakistani and White British children aged 7–11 found higher mean systolic but no evidence of higher diastolic BP in Pakistani children^29^. It is likely that BP in childhood tracks into adulthood^13^ and whilst in the past there has been some inconsistency in reports of ethnic differences in adult BP^31^, more recent evidence from a UK cohort study of European and South Asian men showed that both systolic and diastolic BP were higher among South Asian participants^12^. Our results suggest that ethnic differences in diastolic BP may be present from as early as age 4.

The detailed family social and economic and pregnancy information available for BiB participants, has allowed us to explore potential associations between early life exposures and BP at age 4/5, and whether any associations differ by ethnicity. We found no strong evidence of any associations between family social and economic markers and systolic or diastolic BP in either ethnic group which is consistent with some studies especially those of younger children age 11 and under^9,29^, whereas by adolescent years^10,32^ and adulthood^33,34^ there is evidence for people from more deprived backgrounds having higher BP. It is possible that associations between social and economic environments and BP differ at different stages of the life course, and it will be important to continue to examine the potential influence of these factors as the BiB cohort age.

Consistent with other studies^15,35^, we found that the positive association between maternal early pregnancy BMI and both systolic and diastolic BP in childhood was largely explained by the association of maternal BMI with child BMI and of that with the child’s own BP; here we show that this is the case for both White British and Pakistani children. We found no evidence for an association between maternal gestational smoking and offspring BP at age 4/5 among White British mother-offspring pairs in this cohort, and as smoking was very uncommon among Pakistani origin women, were unable to examine any ethnic difference in this.

In contrast to some other studies^16,17^, we did not find strong evidence for associations between maternal glycaemic traits and offspring BP. Similar to previous studies^36–38^, we identified positive associations between HDP (gestational hypertension) and offspring systolic and diastolic BP at age 4/5, though here we observed that this positive association was stronger in Pakistani compared with White European origin participants. A positive relationship between maternal HDP and offspring BP has been shown by a number of existing studies using mostly White European populations^36,37^ or mixed European and non-European groups^38^, however to our knowledge an ethnic difference in this relationship has not previously been identified. This finding, combined with the early childhood ethnic differences in diastolic BP between White British and Pakistani children in our cohort, support the possibility that South Asian populations may have different BP trajectories to White European populations. Related work with the BiB cohort, which has explored patterns of change in blood pressure in pregnancy, has found that there is a steeper rise in the risk of HDP in later pregnancy (third trimester) among Pakistani origin mothers compared to White British mothers. Similar findings have been reported in a Norwegian multi-ethnic cohort that showed that non-Europeans had the lowest BP in early pregnancy but experienced the greatest increases later in pregnancy^39^. These ethnic differences in BP during pregnancy, together with those we have observed in the women’s offspring at age 4/5 warrant further investigation of ethnic differences in BP trajectories across the life course. Historically, management of BP in adults has mostly been based on white populations^40^ but there are clear ethnic differences in the incidence of cardiovascular disease (CVD)^1,41^. For example, there is some evidence that diastolic BP in particular may be more strongly associated with stroke risk in South Asians compared to Europeans^12^. The differences we have identified in diastolic BP here may therefore be especially important to future risk of adult CVD in this population.

If South Asian populations do have a different BP trajectory and this is evident in early childhood, epigenetic aetiology may be key to explaining this. Population differences in maternal characteristics, obesity prevalence, diet, lifestyle and levels of physical activity may all be important to patterns of BP in childhood^42^ and could present opportunities to modulate the risk of disease in later life. For example, both adherence to a mediterranean diet and high levels of physical activity have been associated with a lower prevalence of cardiometabolic risk markers (including BP) in Southern European children and adolescents (aged 6–14)^43^. There is evidence of lower levels of physical activity^44^ and marked dietary differences^45^ between UK South Asian populations and White British populations and further work is needed to better understand their contribution to ethnic differences in BP.

The strengths of our study are its large size and the availability of a wide range of covariables that allowed us to explore associations of family social and economic and maternal pregnancy exposures with offspring BP, and to adjust for potential confounding factors in these two ethnic groups. Given the age (4/5 years) and the setting (primary school reception class), we were only able to collect one BP measurement and it is possible that a more precise estimate could have been obtained from multiple measurements. However, our approach was to minimise both discomfort and disruption and multiple and any reduction in precision of our estimates is likely to be similar in both ethnic groups. Our results are of two homogenous groups and are not necessarily generalisable to other South Asian or White European groups. Diet and physical activity data were not available for the full BiB cohort which meant we were not able to explore potential diet and activity influences on our results.

In conclusion, we have shown that systolic BP is similar but diastolic BP is on average higher, in Pakistani compared to White British children at age 4/5. Any early identification of those children at risk of hypertension will be key to prevention of later disease. Of the exposures examined here, only HDP (gestational hypertension) seems to be associated with systolic and diastolic BP at age 4/5 and this association was only identified in Pakistani origin mother-offspring pairs. Both these findings suggest that Pakistani populations may have a different BP trajectory compared to White British groups and that this is already evident at age 4/5 years. Further work is needed to confirm or refute this and to understand the importance of diastolic as well as systolic BP to CVD outcomes in South Asian populations.

### Data Availability

Scientists are encouraged and able to use BiB data. Data requests are made to the BiB executive using the form available from the study website http://www.borninbradford.nhs.uk (please click on ‘Science and Research’ to access the form). Guidance for researchers and collaborators, the study protocol and the data collection schedule are all available via the website. All requests are carefully considered and accepted where possible.

## Electronic supplementary material

Associations of social and economic and pregnancy exposures with blood pressure in UK White British and Pakistani children age 4/5 supplementary information
