# Author Correction: Associations of social and economic and pregnancy exposures with blood pressure in UK White British and Pakistani children age 4/5

**DOI:** 10.1038/s41598-018-32318-0

**Published:** 2018-09-26

**Authors:** Jane West, Debbie A. Lawlor, Gillian Santorelli, Paul Collings, Peter H. Whincup, Naveed A. Sattar, Diane Farrar, John Wright

**Affiliations:** 1 Bradford Institute for Health Research, Bradford, UK; 2 MRC Integrative Epidemiology Unit at the University of Bristol, Bristol, UK; 3 Population Health Science, Bristol Medical School, Bristol, UK; 4 Population Health Research Institute, St George’s, University of London, London, UK; 5 Institute of Cardiovascular and Medical Sciences, BHF Glasgow Cardiovascular Research Centre, University of Glasgow, Glasgow, UK

Correction to: *Scientific Reports* 10.1038/s41598-018-27316-1, published online 12 June 2018

The original version of this Article contained errors in Table 2 due to the incorrect coding of the hypertensive disorders of pregnancy (HDP) variable. This error also affected Figure 5 in the original Article, and supplementary Tables 2c, 3c, 4b, 5 and 6c in the Supplementary Information file, which have all been replaced.

As a result, in the Results section,

“By contrast, around twice as many White British women had HDP (both gestational hypertension and pre-eclampsia)”

now reads:

“By contrast, around twice as many White British women had HDP (gestational hypertension) but rates of pre-eclampsia were similar in both groups”

Also in the Results section,

“Associations between HDP (pre-eclampsia) offspring outcomes were also positive but with wide confidence limits that included the null value and reflected the smaller number of participants with pre-eclampsia”

now reads:

“Associations between HDP (pre-eclampsia) offspring outcomes were also positive but with wide confidence limits that mostly included the null value and reflected the smaller number of participants with pre-eclampsia”

These errors have now been corrected in the HTML and PDF versions of this Article, and in the accompanying Supplementary Material.

