# Supplementary material for: Associations of social and economic and pregnancy exposures with blood pressure in UK White British and Pakistani children age 4/5
Source: Sci Rep. 2018 Jun 12;8:8966. doi: 10.1038/s41598-018-27316-1 (PMC5997744; doi:10.1038/s41598-018-27316-1)
Supplement: Supplementary file 1 — Associations of social and economic and pregnancy exposures with blood pressure in UK White British and Pakistani children age 4/5 supplementary information [file 41598_2018_27316_MOESM1_ESM.pdf]

Supplementary Information

Associations of social and economic and pregnancy exposures with blood pressure in UK White British and Pakistani children age 4/5

Jane West
Debbie A Lawlor
Gillian Santorelli
Paul Collings
Peter H Whincup
Naveed A Sattar
Diane Farrar
John Wright

Additional tables (x 13) reporting unadjusted associations and adjusted associations presented numerically (rather than in figures as presented in the manuscript), and additional models referred to but not presented in the manuscript.

Supplementary eTable 1 Distributions of offspring characteristics stratified by ethnicity and sex

Characteristic	All *n=34 68*	White British *n=1644*	Pakistani origin *n=1824*	p-value*	Characteristic
**Child Height (cm)**	*Boys*	108.79 (4.97)	108.48 (4.99)	109.11 (4.93)	0.009
mean (sd)		*n=1670*	*n=822*	*n=848*	
	Girls	108.04 (4.96)	107.70 (4.94)	108.32 (4.97)	0.008
		n=1798	*n=822*	*n=976*	
**Child Weight (kg)**	*Boys*	19.09 (3.02)	19.17 (2.69)	19.02 (3.31)	0.309
mean (sd)		*n=1670*	*n=822*	*n=848*	
	Girls	18.88 (3.13)	19.01 (2.94)	18.77 (3.28)	0.111
		n=1798	*n=822*	*n=976*	
**Child BMI (kg/m^2)**	*Boys*	16.06 (1.69)	16.24 (1.47)	15.89 (1.87)	0.000
mean (sd)		*n=1670*	*n=822*	*n=848*	
	Girls	16.09 (1.77)	16.32 (1.63)	15.91 (1.86)	0.000
		n=1798	*n=822*	*n=976*	
**Child systolic BP**	*Boys*	98.51	98.55 (10.26)	98.47 (10.24)	0.874
mean (sd)		*n=1670*	*n=822*	*n=848*	
	Girls	97.27	97.34 (10.83)	97.21 (11.39)	0.800
		n=1798	*n=822*	*n=976*	
**Child diastolic BP**	*Boys*	60.39 (10.58)	59.54 (10.32)	61.21 (10.76)	0.001
mean (sd)		*n=1670*	*n=822*	*n=848*	
	Girls	62.04 (11.45)	61.48 (10.43)	62.51 (12.23)	0.058
		n=1798	*n=822*	*n=976*	

**Difference between White British and Pakistan*

Supplementary eTable 2a Unadjusted associations between family social and economic exposures and offspring BP at age 4/5 stratified by ethnic group

Measure	Maternal education<br>*Baseline: 5 GCSEs or less*			Housing tenure<br>*Baseline: owning/part owning home*			Receipt of means tested benefits<br>*Baseline: not receiving benefits*		
	White British n=1644	*Pakistani n=1824*	*p value*†	*White British n=1644*	*Pakistani n=1824*	*p value*†	*White British n=1644*	*Pakistani n=1824*	*p value*†
**Systolic mmHg**	0.449 (-0.577, 1.475)	-1.748 (-2.755, -0.739)	0.003	0.089 (-0.941, 1.121)	0.011 (-1.155, 1.176)	0.334	-1.207 (-2.264, -0.151)	0.265 (-.737, 1.267)	0.048
**Diastolic mmHg**	0.368 (-0.644, 1.380)	-1.621 (-2.694, -0.547)	0.009	0.308 (-0.709, 1.326)	-0.263 (-1.50, 0.978)	0.602	-0.319 (-1.363, 0.724)	-0.616 (-1.682, 0.450)	0.699

Values are differences in means (95% CI) of outcome per maternal exposure unit or category
† interaction p-value, difference between White British and Pakistani

Supplementary eTable 2b
Unadjusted associations between pregnancy exposures (BMI and smoking) and offspring BP at age 4/5 stratified by ethnic group

**Measure**	**Maternal BMI** *Difference in means per 5 kg/m²*			**Maternal smoking in pregnancy** *Baseline: no smoking*		
	White British n=1644	*Pakistani n=1824*	*p value†*	*White British n=1644*	*Pakistani n=1824*	*p value†*
**Systolic mmHg**	0.308 (-0.121, 0.736)	0.666 (0.203, 1.129)	0.265	-0.184 (-1.268, 0.899)	0.023 (-2.675, 2.720)	0.887
**Diastolic mmHg**	0.067 (-0.355, 0.489)	0.339 (-0.154, 0.832)	0.412	-0.292 (-1.361, 0.777	1.089 (-1.781, 3.960)	0.360

Values are differences in means (95% CI) of outcome per exposure unit or category, maternal BMI is difference in means per 5kg/m²
†interaction p-value, difference between White British and Pakistani

Supplementary eTable 2c
Unadjusted associations between pregnancy exposures (glycaemia and HDP) and offspring BP at age 4/5 stratified by ethnic group

Measure	**Fasting glucose** *Difference in means per 1mmol/L*			**Post-load glucose** *Difference in means per 1mmol/L*			**GDM** *Baseline: no GDM*			**HDP: gestational hypertension** *Baseline: no gestational hypertension*			**HDP: pre-eclampsia** *Baseline: pre-eclampsia*		
	White British n=1644	*Pakistani n=1824*	*p value*†	*White British n=1644*	*Pakistani n=1824*	*p value*†	*White British n=1644*	*Pakistani n=1824*	*p value*†	*White British n=1644*	*Pakistani n=1824*	*p value*†	*White British n=1644*	*Pakistani n=1824*	*p value*†
**Systolic mmHg**	1.203 (0.011, 2.396)	0.981 (0.104, 1.857)	0.769	0.422 (0.028, 0.816)	0.278 (-0.032, 0.588)	0.575	1.189 (-1.186, 3.563)	0.979 (-0.702, 2.659)	0.888	1.359 (-0.271, 2.988)	3.181 (0.759, 5.602)	0.219	2.566 (-0.568, 5.699)	3.905 (0.617, 7.193)	0.563
**Diastolic mmHg**	-0.032 (-1.209, 1.145)	0.667 (-0.267, 1.601)	0.371	0.141 (-0.249, 0.530)	0.196 (-0.134, 0.526)	0.834	0.687 (-1.655, 3.029)	0.851 (-.938, 2.639)	0.915	0.355 (-1.254, 1.964)	3.464 (0.883, 6.045)	0.042	1.597 (-1.497, 4.691)	1.186 (-2.318, 4.689)	0.863

Values are differences in means (95% CI) of outcome per exposure unit or category
†interaction p-value, difference between White British and Pakistani

Supplementary eTable 3a Adjusted associations of family social and economic exposures and offspring BP at age 4/5 stratified by ethnic group (Model 1*)

**Measure**	**Maternal education** *Baseline: 5 GCSEs or less*			**Housing tenure** *Baseline: owning/part owning home*			**Receipt of means tested benefits** *Baseline: not receiving benefits*		
	White British n=1644	*Pakistani n=1824*	*p value*†	*White British n=1644*	*Pakistani n=1824*	*p value*†	*White British n=1644*	*Pakistani n=1824*	*p value*†
**Systolic mmHg**	0.419 (-0.600, 1.439)	-1.753 (-2.759, -0.749)	0.003	0.112 (0.912, 1.137)	0.132 (-1.033, 1.297)	0.945	-1.196 (-2.246, -0.145)	0.329 (-0.669, 1.329)	0.038
**Diastolic mmHg**	0.414 (-0.595, 1.421)	-1.577 (-2.649, -0.503)	0.009	0.321 (-0.693, 1.334)	-0.226 (-1.469, 1.017)	0.508	-0.376 (-1.416, 0.664)	-0.602 (-1.667, 0.464)	0.761

**Model 1: Adjusted for sex; age at measurement*
Values are differences in means (95% CI) of outcome per maternal exposure unit or category
†interaction p-value, difference between White British and Pakistani

Supplementary eTable 3b Adjusted associations of pregnancy exposures (BMI and smoking) and offspring BP at age 4/5 stratified by ethnic group (Model 1*)

**Measure**	**Maternal BMI** ***Difference in means per 5 kg/m^2***			**Maternal smoking in pregnancy** ***Baseline: no smoking***		
	White British n=1644	*Pakistani n=1824*	*p value†*	*White British n=1644*	*Pakistani n=1824*	*p value†*
**Systolic mmHg**	0.314 (-0.112, 0.740)	0.678 (0.217, 1.139)	0.248	-0.172 (-1.249, 0.906)	-0.022 (-2.711, 2.667)	0.888
**Diastolic mmHg**	0.101 (-0.319, 0.523)	0.333 (-0.159, 0.826)	0.476	-0.238 (-1.304, 0.827)	1.149 (-1.718, 4.018)	0.360

Model 1: Adjusted for sex; age at measurement

Values are differences in means (95% CI) of outcome per maternal exposure unit or category, maternal BMI is difference in means per 5kg/m^2

†interaction p-value, difference between White British and Pakistani

Supplementary eTable 3c Adjusted associations of pregnancy exposures (glycaemia and HDP) and offspring BP at age 4/5 stratified by ethnic group (Model 1*)

Measure	**Fasting glucose** *Difference in means per 1mmol/L*			**Post-load glucose** *Difference in means per 1mmol/L*			**GDM** *Baseline: no GDM*			**HDP: gestational hypertension** *Baseline: no gestational hypertension*			**HDP: pre-eclampsia** *Baseline: no pre-eclampsia*		
	White British n=1644	*Pakistani n=1824*	*p value*†	*White British n=1644*	*Pakistani n=1824*	*p value*†	*White British n=1644*	*Pakistani n=1824*	*p value*†	*White British n=1644*	*Pakistani n=1824*	*p value*†	*White British n=1644*	*Pakistani n=1824*	*p value*†
**Systolic mmHg**	1.114 (-0.072, 2.299)	0.955 (0.079, 1.830)	0.802	0.404 (0.013, 0.797)	0.268 (-0.041, 0.578)	0.570	1.037 (-1.325, 3.399)	0.872 (-0.804, 2.548)	0.880	1.299 (-0.321, 2.919)	3.051 (0.636, 5.466)	0.240	2.459 (-0.658, 5.575)	3.909 (0.636, 5.466)	0.529
**Diastolic mmHg**	-0.026 (-1.199, 1.148)	0.624 (-0.310, 1.559)	0.411	0.140 (-.248, 0.528)	0.182 (-0.149, 0.512)	0.886	0.583 (-1.753, 2.919)	0.849 (-0.938, 2.638)	0.869	0.446 (-1.158, 2.049)	3.508 (0.929, 6.087)	0.043	1.812 (-1.271, 4.896)	1.154 (-2.347, 4.654)	0.792

**Model 1: Adjusted for sex; age at measurement*
Values are differences in means (95% CI) of outcome per maternal exposure unit or category
†interaction p-value, difference between White British and Pakistani

Supplementary eTable 4a Adjusted associations of pregnancy exposures (BMI and smoking) and offspring BP at age 4/5 stratified by ethnic group (Model 2*)
(For Social and economic variables, model 1 and 2 are the same)

Measure	Maternal BMI** *Difference in means per 5 kg/m²*			Maternal smoking in pregnancy *Baseline: no smoking*		
	White British n=1644	*Pakistani n=1824*	*p value†*	*White British n=1644*	*Pakistani n=1824*	*p value†*
**Systolic mmHg**	0.261 (-0.174, 0.695)	0.564 (0.078, 1.049)	0.309	0.262 (-0.919, 1.442)	-0.030 (-2.728, 2.667)	0.974
**Diastolic mmHg**	0.024 (-0.407, 0.455)	0.305 (-0.214, 0.824)	0.468	-0.009 (-1.179, 1.162)	1.131 (-1.749, 4.010)	0.390

*Model 2: Adjusted for sex; age at measurement; maternal age; parity; maternal education; family housing tenure; family receipt of benefits
**Additionally adjusted for smoking in pregnancy
Values are differences in means (95% CI) of outcome per maternal exposure unit or category, maternal BMI is difference in means per 5kg/m²
†interaction p-value, difference between White British and Pakistani

Supplementary eTable 4b Adjusted associations of pregnancy exposures (glycaemia and HDP) and offspring BP at age 4/5 stratified by ethnic group (Model 2*)

**Measure**	**Fasting glucose** *Difference in means per 1mmol/L*			**Post-load glucose** *Difference in means per 1mmol/L*			**GDM** *Baseline: no GDM*			**HDP: gestational hypertension** *Baseline: no gestational hypertension*			**HDP: pre-eclampsia** *Baseline: no pre-eclampsia*		
	White British n=1644	*Pakistani n=1824*	*p value†*	*White British n=1644*	*Pakistani n=1824*	*p value†*	*White British n=1644*	*Pakistani n=1824*	*p value†*	*White British n=1644*	*Pakistani n=1824*	*p value†*	*White British n=1644*	*Pakistani n=1824*	*p value†*
**Systolic mmHg**	0.894 (-0.307, 2.095)	0.838 (-0.059, 1.735)	0.806	0.292 (-0.108, 0.693)	0.216 (-0.107, 0.538)	0.524	0.599 (-1.778, 2.977)	0.683 (-1.024, 2.390)	0.836	1.078 (-0.544, 2.711)	3.104 (0.678, 5.529)	0.253	2.301 (-0.825, 5.427)	4.043 (0.762, 7.324)	0.521
**Diastolic mmHg**	-0.243 (-1.435, 0.949)	0.573 (-0.385, 1.531)	0.385	0.070 (-0.327, 0.467)	0.156 (-0.189, 0.500)	0.885	0.321 (-2.037, 2.679)	0.761 (-1.061, 2.583)	0.865	0.355 (-1.265, 1.975)	3.355 (0.763, 5.948)	0.049	1.854 (-1.249, 4.957)	1.101 (-2.406, 4.609)	0.751

**Model 2: : Adjusted for sex; age at measurement; maternal age; parity; smoking in pregnancy; maternal education; family housing tenure; family receipt of benefits*
Values are differences in means (95% CI) of outcome per maternal exposure unit or category
†interaction p-value, difference between White British and Pakistani

Supplementary eTable 5 Adjusted associations of pregnancy exposures (glycaemia and HDP) and offspring BP at age 4/5 stratified by ethnic group (Model 3*)
(For Social and economic variables, model 1, 2 and 3 are the same; for family variables models 2 and 3 are the same)

**Measure**	**Fasting glucose** ***Difference in means per 1mmol/L***			**Post-load glucose** ***Difference in means per 1mmol/L***			**GDM** ***Baseline: no GDM***			**HDP: gestational hypertension** ***Baseline: no gestational hypertension***			**HDP: pre-eclampsia** ***Baseline: no pre-eclampsia***		
	White British n=1644	*Pakistani n=1824*	*p value†*	*White British n=1644*	*Pakistani n=1824*	*p value†*	*White British n=1644*	*Pakistani n=1824*	*p value†*	*White British n=1644*	*Pakistani n=1824*	*p value†*	*White British n=1644*	*Pakistani n=1824*	*p value†*
**Systolic mmHg**	0.749 (-0.504, 2.003)	0.613 (-0.314, 1.539)	0.908	0.259 (-0.147, 0.665)	0.155 (-0.172, 0.482)	0.557	0.498 (-1.886, 2.881)	0.408 (-1.316, 2.131)	0.810	0.914 (-0.773, 2.601)	2.818, (0.372, 5.265)	0.231	2.169 (-0.976, 5.314)	3.685 (0.379, 6.990)	0.526
**Diastolic mmHg**	-0.285 (-1.529, 0.959)	0.459 (-0.531, 1.450)	0.355	0.068 (-0.335, 0.472)	0.124 (-0.226, 0.474)	0.870	0.313 (-2.052, 2.679)	0.619 (-1.222, 2.462)	0.875	0.377 (-1.298, 2.051)	3.218 (0.601, 5.835)	0.048	1.871 (-1.251, 4.993)	0.929 (-2.607, 4.465)	0.749

**Model 3: Adjusted for sex; age at measurement; maternal BMI; maternal age; parity; smoking in pregnancy; maternal education; family housing tenure; family receipt of benefits*
Values are differences in means (95% CI) of outcome per maternal exposure unit or category
†interaction p-value, difference between White British and Pakistani

Supplementary eTable 6a Adjusted associations of family social and economic exposures and offspring BP at age 4/5 stratified by ethnic group (Model 4*)

Measure	Maternal education *Baseline: 5 GCSEs or less*			Housing tenure *Baseline: owning/part owning home*			Receipt of means tested benefits *Baseline: not receiving benefits*		
	White British n=1644	*Pakistani n=1824*	*p value*†	*White British n=1644*	*Pakistani n=1824*	*p value*†	*White British n=1644*	*Pakistani n=1824*	*p value*†
**Systolic mmHg**	0.651 (-0.349, 1.652)	-1.591 (-2.569, -0.613)	0.003	-0.069 (-1.074, 0.935)	0.033 (-1.100, 1.167)	0.945	-1.360 (-2.389, -0.331)	0.272 (-0.699, 1.244)	0.038
**Diastolic mmHg**	0.543 (-0.459, 1.547)	-1.461 (-2.522, -0.400)	0.009	0.219 (-0.789, 1.227)	-0.297 (-1.525, 0.931)	0.508	-0.468 (-1.501, 0.566)	-0.643 (-1.696, 0.410)	0.761

**Model 4: Adjusted for sex; age at measurement; maternal age; parity; smoking in pregnancy; maternal education; family housing tenure; family receipt of benefits; child BMI at BP measurement*
Values are differences in means (95% CI) of outcome per maternal exposure unit or category
†interaction p-value, difference between White British and Pakistani

Supplementary eTable 6b Adjusted associations of pregnancy exposures (BMI and smoking) and offspring BP at age 4/5 stratified by ethnic group (Model 4*)

**Measure**	**Maternal BMI**** *Difference in means per 5 kg/m²***			**Maternal smoking in pregnancy** *Baseline: no smoking*		
	White British n=1644	*Pakistani n=1824*	*p value†*	*White British n=1644*	*Pakistani n=1824*	*p value†*
**Systolic mmHg**	-0.270 (-0.713, 0.172)	-0.228 (-.726, 0.271)	0.863	0.242 (-0.913, 1.397)	-1.367 (-4.003, 1.269)	0.392
**Diastolic mmHg**	-0.279 (-0.725, 0.167)	-0.265 (-0.805, 0.276)	0.858	-0.020 (-1.184, 1.143)	0.186 (-2.674, 3.045)	0.753

**Model 4: Adjusted for sex; age at measurement; maternal age; parity; maternal education; family housing tenure; family receipt of benefits; child BMI at BP measurement*
***Additionally adjusted for smoking in pregnancy*
Values are differences in means (95% CI) of outcome per maternal exposure unit or category, maternal BMI is difference in means per 5kg/m²
†interaction p-value, difference between White British and Pakistani

Supplementary eTable 6c Adjusted associations of pregnancy exposures (glycaemia and HDP) and offspring BP at age 4/5 stratified by ethnic group (Model 4*)

Measure	Fasting glucose** *Difference in means per 1mmol/L*			Post-load glucose** *Difference in means per 1mmol/L***			GDM** *Baseline: no GDM*			HDP: gestational hypertension** *Baseline: no gestational hypertension*			HDP: pre-eclampsia** *Baseline: no pre-eclampsia*		
	White British n=1644	*Pakistani n=1824*	*p value†*	*White British n=1644*	*Pakistani n=1824*	*p value†*	*White British n=1644*	*Pakistani n=1824*	*p value†*	*White British n=1644*	*Pakistani n=1824*	*p value†*	*White British n=1644*	*Pakistani n=1824*	*p value†*
**Systolic mmHg**	0.876 (-0.350, 2.103)	0.598 (-0.305, 1.500)	0.576	0.286 (-0.111, 0.684)	0.119 (-0.199, 0.438)	0.314	0.716 (-1.617, 3.049)	0.263 (-1.416, 1.941)	0.546	0.981 (-0.671, 2.632)	2.386 (0.002, 4.769)	0.463	2.173 (-0.905, 5.251)	4.130 (0.910, 7.350)	0.440
**Diastolic mmHg**	-0.213 (-1.449, 1.023)	0.448 (-0.531, 1.428)	0.514	0.084 (-0.316, 0.485)	0.098 (-0.248, 0.444)	0.926	0.438 (-1.912, 2.788)	0.516 (-1.305, 2.336)	0.945	0.414 (-1.248, 2.078)	2.909 (0.320, 5.497)	0.091	1.874 (-1.227, 4.975)	1.246 (-2.251, 4.743)	0.807

**Model 4: Adjusted for sex; age at measurement; maternal age; parity; smoking in pregnancy; maternal education; family housing tenure; family receipt of benefits; child BMI at BP measurement*
***Additionally adjusted for maternal BMI*
Values are differences in means (95% CI) of outcome per maternal exposure unit or category
†interaction p-value, difference between White British and Pakistani
